# Obstetrics

**Published:** 1878-01

**Authors:** 


### II. Obstetrics.

Cephalotripsy.—M. Gillette. (*L'Union Médicale,* Oct. 2, 1877. M. Gillette communicated to the *Société de Médecine de Paris,* session of July 14, 1877, a case of cephalotripsy which he had just performed upon an Italian hunchback, in whom the antero-posterior diameter of the pelvis was narrowed at least three centimeters. The pregnancy was at full term, and the child was well formed. M. Gillette hesitated for some time, asking himself, before proceeding to crush the child's head, if it was not better, in place of sacrificing the child, to perform the Cæsarean operation in this deformed dwarf, incapable of child-bearing, and of doubtful utility to society. He conformed, however, to the general rule, and performed cephalotripsy—with regret, however.

M. Dubuc asked what was the fate of the woman after the operation. He asked this with the more interest, because in two cases which he remembered, cephalotripsy performed upon two rachitic women at *La Maternité,* was followed by the death of both, and the autopsy showed that the uterus was considerably torn.

M. Gillette observed that he had only been called accidentally to the patient, who was not in his service, and had lost sight of her the day after the operation. He could say, however, that the belly was not painful on that day, though the uterus was still large, and the pulse 160.

M. Leblond would not have hesitated between Cæsarean section and cephalotripsy in the case before them. The first of these operations is much less dangerous than is generally supposed; and the second, on the contrary, eight times out of ten, caused the death of the mother. Moreover, if the Cæsarean operation has not, until now, been more successful, it is due to certain things neglected in the usual method of operating. For example, after extraction of the child, one should proceed as in ovariotomy, cleansing the parts carefully, and the edges of the uterine wound should be brought together by means of elastic sutures. Finally, what seems to prove the advantage of these precautions better than any theory, is the cases in which some operators have, without intending it, when beginning an ovariotomy, been led by circumstances, to perform on the same woman the Cæsarean operation. It would be easy to find examples of this kind in gynecological records, and the successes obtained are already very encouraging.

M. de Beaurais remarked that whatever the perfection of arrangements in the Cæsarean operation, it required still more, a certain skill in operations of this kind; it is necessary also to have skillful assistants. Now, as it is not always possible to realize at will these diverse conditions, it may be conceived how many physicians recoil before the idea of active intervention, which brings with it always a high degree of responsibility.

M. Durasiez added, that if the Cæsarean operation was not oftener performed, it was because the necessity was avoided in a large number of women, by provoking in time, premature delivery.

M. Camuset believes that there is good reason to resort, by preference, to this latter means of intervention; for, in a case he had seen of a little dwarf, who had reached full term, and was already in labor, the Cæsarean operation was promptly followed by death.

M. E. R. Perrin thinks that although ovariotomy and Cæsarean section are two quite dissimilar operations; the success which the former has achieved, should remove the antipathy which Paris obstetricians have for the Cæsarean operation, only the operation should not be done as it usually is, after the patient is exhausted by a prolonged and fruitless labor, and after several applications of the forceps and repeated tractions, which have been, at least, useless. If, on the contrary, the Cæsarean operation be performed at the beginning of labor, always with the authority of a sufficient experience, the chances of success would be greater, for the patient would be free from the traumatisms she would have received from a more tardy intervention.

As to the chances of hemorrhage which the Cæsarean operation made in a manner prematurely, might cause the patient, one could not deny them; but what were they compared to the usual success of late operations?

Finally, are not the cases published from time to time, of non-fatal accidental deliveries, the result of violent injuries of the anterior abdominal region, of a nature to decide the most hesitating?

M. Gillette was very glad to see the general preference of his colleagues for the Cæsarean operation over cephalotripsy. If another case of the kind he had brought before them, should present itself, he would not hesitate in performing immediately, the Cæsarean operation without previously applying the forceps.

L. W. C.

Ano-Pelvian Version.—M. Guéniot. (*L' Union Médicale*, Oct, 4, 1877.) At the session of the *Académie de Médecine*, of Oct. 2, 1877, M. Guéniot read a paper on a "method of version applicable in difficult cases," which the author calls the *ano-pelvian* method.

According to M. Guéniot, circumstances are not lacking where the accoucheur, although well aware of the difficulties and dangers of version, yet feels obliged to resort to this operation.

The most common case, without doubt, is that of a presentation of the trunk with a complication of uterine spasm, where derotomy and evisceration of the fœtus have been judged impracticable, or recognized as inefficacious. In such cases the use of the ano-pelvian process is perfectly indicated.

This process consists essentially:

*First.* In aiding, by the weight of the body, the insertion of of the hand to the fundus of the womb.

*Second.* In taking as a *point d'appui* for the traction to be made upon the fœtus, the pubic arch or the sacro-coccygian process, by the aid of a finger hooked in the rectum.

*Third.* In following, finally, the usual rule for podalic version.

According to this method of operating, after having introduced his hand into the vagina, the accoucheur advances his body into contact with the elbow with which he operates; then he presses with the body, with greater or less force, upon the olecranon process and posterior surface of the arm. The forearm thus pushed forward at the will of the operator, carries, in a mechanical manner, the hand into the uterus, almost without fatigue, in search of the breech. As soon as this is found—which is ordinarily much easier to do than to reach a foot or a knee—the finger is introduced into the rectum and used as a hook to make continuous traction, either on the pubis or on the sacrum, bringing the pelvis of the child into the cavity of the mother's pelvis.

The author sums up the advantages of the ano-pelvian method thus:

*First.* The pelvis of the child is generally easier to find than the feet.

*Second.* The hold which the pelvis or scrotum affords is of the firmest, and does not allow slipping.

*Third.* Traction being direct, the force used is all utilized.

*Fourth.* Whatever the direction of the traction, toward the dorsal or toward the abdominal region of the fœtus, its evolution may be effected.

*Fifth.* Finally, when the operator has failed by the podalic method, the ano-pelvian method still allows of bringing the version to a favorable termination.

M. Guéniot, who has used this method for ten years, is the first to publish it, though others have had recourse accidentally to direct traction on the breech, without attaching to it the merit to which it is entitled. L. W. C.

Normal Labor During Extra-Uterine Pregnancy. (*Jour. de Méd.*, November, 1877.) M. Labatut reports in the *Revue de Littérature Médicale*, the following case:

A woman had had a previous normal labor, and two years later had all the signs of pregnancy. At the end of five months, after progressive development of the abdomen, she had violent pains, but without result. After their cessation, she was sick for six months. Menstruation then reappeared, and the patient enjoyed tolerable health; the tumor had subsided, and the abdominal pains had disappeared.

Five years later, a new suppression of the menses, attended with digestive troubles, took place. The patient, who was then 41 years of age, attributed these symptoms to "change of life." After several months, she was examined by Mme. Rampin, a midwife of Toulon, who diagnosticated pregnancy at the ninth month. She determined, at the same time, also the presence of a voluminous tumor in the right side, which, after hearing the history of the patient, she attributed to an extra-uterine pregnancy. Fifteen days after the examination, the woman was delivered naturally, after a labor of three hours, of a living child. After the labor, the midwife examined carefully the remaining abdominal tumor, due to the extra-uterine pregnancy.

The woman lived two years, and died in March, 1877, at the hospital at Toulon, of pulmonary tuberculosis.

At the autopsy, there was found in the right Fallopian tube, a fœtus at term, macerated, and enveloped in a thick pouch.

The case was, then, one of tubal pregnancy, dating back seven years; in spite of its presence, there had been normal conception and delivery. L. W. C.

THE PROPHYLACTIC TREATMENT OF PLACENTA PRÆVIA. T. Gaillard Thomas (*Am. Practitioner*, May, 1877), earnestly advocates the induction of premature labor, after the period of viability of the child, as the only known method by which the evils attending upon the last three months of utero-gestation, in this complication, can be avoided.

Fortunately, this condition is usually revealed by reliable premonitions, so that the uterus may be emptied of its contents before the vital forces of mother and child are exhausted by hemorrhage. By this means, the physician would be in attendance at the moment of cervical dilation, and consequently the moment of danger, he would be able, by hydrostatic pressure, to control hemorrhage, while at the same time the dilation of the cervix may be rapidly accomplished.

The only objection that can be urged is, that a child of less than nine months' intra-uterine life, does not have as good a prospect of life as one arrived at full term; and this is invalidated by the fact that an eight months' child, depending upon the pulmonary respiration, has a brighter prospect for life than one in the uterus depending for aëration of its blood upon a crippled and bleeding placenta; while for the mother the safety is incomparably greater.

To those physicians who have been satisfied with the tampon until version has become practicable, and who, relying upon these excellent and efficient means, set their faces against the innovation here advocated, he urges a thoughtful consideration of statistics; accepting those of Simpson, Read and Trask in placenta prævia, the prognosis for the mother is about as grave as that of patients submitted to the capital operation of ovariotomy. For the child it is much graver. In view of these facts, he insists that the claims of any means which offers immunity, to any decided degree, from the ordeal of so dangerous a parturition and labor, should be most carefully weighed before being thrown aside. M. E. K.

